# Cigarette retailer density around schools and neighbourhoods in Bali, Indonesia: A GIS mapping

**DOI:** 10.18332/tid/110004

**Published:** 2019-07-05

**Authors:** Putu A. S. Astuti, Ketut H. Mulyawan, Susy K. Sebayang, Ni Made D. Kurniasari, Becky Freeman

**Affiliations:** 1 Department of Public Health and Preventive Medicine, Universitas Udayana, Denpasar, Indonesia; 2 The University of Sydney School of Public Health, Sydney, Australia; 3 Udayana Center for NCDs, Tobacco Control and Lung Health (Central), Universitas Udayana, Denpasar, Indonesia; 4 Department of Biostatistics and Population Studies, Universitas Airlangga, Banyuwangi, Indonesia; 5 Prevention Research Collaboration (PRC), Charles Perkins Centre, The University of Sydney, Sydney, Australia

**Keywords:** cigarette retailers, retailer density, tobacco control, GIS, Indonesia

## Abstract

**INTRODUCTION:**

The presence and density of tobacco retailers is associated with the perception of high availability of cigarettes and ease of purchase. Indonesia is the second largest cigarette market in the world with an increasing smoking rate among young people aged 10–18 years. Our study aims to assess density of cigarette outlets in neighbourhoods and around schools, and to evaluate correlation between retailer proximity to schools and retailer selling practices.

**METHODS:**

We conducted a geographical mapping and then an audit survey of 1000 randomly selected cigarette retailers in Denpasar, Bali, Indonesia. We measured neighbourhood retailer density, and retailer proximity to schools. We linked the coordinate data to the audit data to assess the association between retailer distance from schools with likelihood of selling tobacco to young people and selling single cigarette sticks.

**RESULTS:**

We mapped 4114 cigarette retailers in Denpasar, the most common type was a kiosk, 3199 (77.8%), followed by mini market/convenience stores, 606 (14.7%). Retailer density was 32.2/km^2^ and 4.6/1000 population. We found that 37 (9.7 %) of the 379 schools in Denpasar have at least one cigarette retailer within a 25 m radius and 367 (96.8%) within a 250 m radius. Of the 485 audited retailers within a 250 m radius of a school, 281 (57.9%) admitted selling cigarettes to young people and 325 (67.0%) sold cigarettes as single sticks. Cigarette retailers were less likely to sell cigarettes to young people based on distance from schools, but this was only significant at the furthest distance of more than 500 m from schools.

**CONCLUSIONS:**

In an unregulated retailer setting such as Indonesia, cigarette retailers are ubiquitous and selling to young people is commonplace. The Indonesian government should enforce the prohibition on selling to young people and should regulate cigarette retailers to reduce youth access to cigarettes.

## INTRODUCTION

Globally, tobacco control measures focusing on reducing the demand for tobacco products have been progressing well, yet limited action has been taken on the supply side^1,2^. Tobacco retailers are essential players in the tobacco industry marketing chain, where the four items of marketing — product, place, price, and promotion — occur in one convenient place. Regulating tobacco retailers will contribute to both reducing access to cigarettes and environmental cues to smoking, which in turn will accelerate the denormalisation of tobacco use^3,4^.

The presence and density of tobacco retailers are associated with the perception of high availability of cigarettes and ease of purchase^5^ and with the notion that smoking is common and acceptable^6^. Higher tobacco outlet density around a neighbourhood or a school is correlated with higher purchase attempts^7^, smoking frequency^8,9^, the number of cigarettes smoked in past 7 days^10^, and lifetime smoking^5^. Cigarette retailer density is also a barrier to successful smoking cessation^11^ and increases susceptibility to future smoking among non-smokers^7,11,12^.

Tobacco retailer density is also associated with a disparity in smoking prevalence. Higher outlet density has been observed in areas that are home to a greater proportion of minority populations, and in neighbourhoods with lower socioeconomic status and higher numbers of disadvantaged populations^13-17^. Tobacco outlets are more prevalent in areas with more minors^14^ and in areas closer to schools^17^. Policies that ban sales of tobacco products in areas around schools^18^ and the introduction of a retailer licensing scheme would contribute to a significant reduction in retailer density^19^, which in turn may reduce youth access to cigarettes.

Indonesia is the second largest cigarette market in the world, with an overall retail volume of 316.1 billion sticks sold per year in 2016^20^. Cigarette retailers in Indonesia are ubiquitous, with most food stores and small kiosks selling cigarettes. PT HM Sampoerna, majority owned and operated by Philip Morris International, and the biggest tobacco company in Indonesia, distributes their products through approximately 2.4 million points-of-sale throughout Indonesia^21^.

To date, the Indonesian government has adopted only a small number of WHO recommended regulations^22^ to reduce the demand for tobacco products and has not endorsed any approaches to curb tobacco supply beyond the weakly enforced ban on sales to minors in the national tobacco control regulation (Peraturan Pemerintah/PP No 109/2012)^23^. According to the Global Youth Tobacco Survey (GYTS) 2014, 19.4% of Indonesian junior high school students (aged 13–15 years) were current smokers, accounting for 22.4 million students. Almost two-thirds (64.5%) of the student smokers freely purchased their cigarettes, and three-quarters (75%) of them bought them as single sticks. Additionally, approximately 5.2 million (4.5%) of non-smoking students were susceptible to future smoking^24^. Smoking prevalence among young people, aged 10–18 years, increased from 7.2% in 2013 to 9.2% in 2018^25^, moving in the opposite direction of the government’s target of 5.4% in 2019. The current situation of only partially adopted and poorly enforced measures is not enough to control the rising epidemic of tobacco use. Supply-side policies such as comprehensive tobacco retailer regulation may help accelerate tobacco control progress in Indonesia.

Some countries and jurisdictions have adopted tobacco retailer regulation to help control supply and to reduce youth access and smoking prevalence^26-28^. Assessing the magnitude of cigarette retailing is a necessary first step towards the adoption of any potential regulation. Geographical mapping has been applied to determine the distribution of cigarette retailers and to evaluate retailer compliance with licensing schemes and other regulations^29,30^. Mapping provides the spatial distribution of retailers, which can serve as practical evidence for policy development or evaluation following the implementation of future regulations.

Denpasar is the capital city of Bali Province. Bali was the first Indonesian province to adopt a provincial smoke-free regulation in 2011. The government of the City of Denpasar also enacted a smoke-free law in 2013^31^, and adopted outdoor tobacco advertisement restrictions in 2015^32^. The smoke-free law implemented in Denpasar prohibits cigarette sales and promotions within schools; however, cigarette retailers remain highly prevalent close to schools^33,34^. The retail outlets are adorned with cigarette advertisements^33^ and visible pack displays^35^ that expose children to positive tobacco images^36^. To date, there is no registry or mapping of cigarette retailers available in Denpasar. Mapping cigarette retailers around schools and neighbourhoods will provide important information to policymakers about the ease of access to cigarettes by young people. This information could then also guide policy responses needed to reduce both access to cigarettes and exposure to cigarette ads. Our study aims to assess the distribution and density of cigarette outlets in neighbourhoods and to assess the spatial distribution within proximity to schools in Denpasar. We also explored the correlation between retailer proximity to schools and the likelihood of selling cigarettes to young people and the sale of single cigarettes.

## METHODS

### Study setting

The study was conducted in the City of Denpasar in the period December 2017 – January 2018. Denpasar is categorised as a ‘big city’ based on its population size. In 2016, the registered population^37^ was 893700, and the population density was approximately 7022 people/km^2^. Denpasar’s area is 127.78 km^2^, divided into four sub-districts with 43 kelurahan/desa (neighbourhoods), with an average size of 2.97 km^2^ (range: 0.33–9.71 km2)^37^. The Kelurahan/desa is the neighbourhood mapping unit for our study.

In 2017, the total number of schools in Denpasar was 380, with approximately 158114 students^38^. We included all primary, junior and senior high schools in the mapping. In the Indonesian education system, primary, junior and senior high school education are generally held in separate institutions and different locations. In Denpasar, one junior and one senior high school operate in the same place, represented by one coordinate and recorded only once for our mapping, resulting in a total of 379 school locations.

### Data collection procedure

We conducted the study in two stages: 1) Geographical mapping of cigarette retailers and schools, and 2) Audit survey with 1000 randomly selected retailers mapped in the 1st stage, which included observation of tobacco promotion, digital photo taking and structured questionnaire survey with the retailers, described in detail elsewhere^35^.

Cigarette outlet coordinates were collected by four pairs of trained enumerators (recent graduates from the Bachelor of Public Health degree course) after receiving 1.5 days of in-class and field training. Each pair was in charge of covering one sub-district and collecting retailer data by motorbike. We tracked the enumerators’ routes with My Tracks App to ensure that they included all areas of the city. We excluded small alleys with only single motorbike access. We also excluded bars, hotels and restaurants from the mapping as these types of retailers usually only sell primarily to their customers/patrons. Enumerators submitted their coordinates and the route was tracked online to data manager (KHM) on a daily basis. The enumerators identified cigarette retailers by either seeing a cigarette display, or seeing cigarettes being purchased, or asking the retailer directly when neither was observed. The time of observation was between 9 am – 6 pm to match the opening hours of most retailers. The enumerators filled out an electronic checklist on Open Data Kit (ODK)^35^ embedded in their mobile phone. It included the store name, type of store, and coordinate location. The global positioning system (GPS) coordinates were taken with 7 m precision, which was automatically validated within the ODK system. KHM cross-checked the coordinates in the geo-coordinate precision column when the data were submitted and randomly checked 100 submitted retailer coordinates using Google Map Street View as a reliability check of the location and retailer attributes. For the school coordinates, we retrieved the school addresses from the Ministry of Education website and geo-coded these with Geographic Information System (GIS) to determine the coordinates.

### Measures

#### Retailer density and proximity to schools

All coordinate data were transferred from ODK to ArcGIS 10.5. We measured retailer density in neighbourhoods and around schools by the following:

1. Neighbourhood-based measures included number of retailers/1000 population, number of retailers/km^2^, number of retailers/km^2^ of occupied land for houses or buildings (excluding areas for farming and open fields), number of retailers per kelurahan/desa, and number of retailers within a certain radius (50, 100, 250 and 500 m) of another retailer. We also assessed proximity to other outlets, defined as the distance between a tobacco retailer and the next closest outlet.
2. School-based measures included the number of outlets within a 25, 50, 100, 250 and 500 m radius from the schools and the number of schools with at least one cigarette outlet within these radii. We also measured the proximity (distance) of a school to the closest outlet.

We used the point distance tool on the ArcGIS 10.5 to calculate the distance from a school to the nearest cigarette retail outlet, distance from one outlet to another, and the number of retail outlets within each radius.

#### School characteristics

School characteristics included in the analysis were school level (primary, junior and senior high school) and school type (public and private).

#### Population density

We calculated population density for each kelurahan/desa, as the total population divided by the size of kelurahan/desa in km^2^. Then, we categorised them into four equal groups based on the GIS quantile classification method^39^ and assigned a colour gradation for each on the Choropleth map.

#### Retailer selling practices

We linked the coordinate data with the retailer audit survey data on selling cigarettes to young people and selling single cigarettes. Cigarette selling practices were obtained through retailer responses to a face-toface survey conducted during the audit. For the audit survey, we (NMDK) conducted a reliability check of 25 randomly selected retailers.

### Statistical analysis

We conducted a descriptive analysis, including the proportion of retailer types and the median number of outlets within a certain radius from schools or another outlet. We compared the proportion of schools that had at least one cigarette outlet in each radius based on school level and type using a chi-squared test. We examined the retailer behaviour of selling single cigarettes and selling to young people based on retailer distance from the schools using logistic regression, adjusted for school level, school type, and retailer types. For this analysis, variable distance from schools was categorised into four groups: ≤100, 100.1–250, 250.1–500 and >500 m. We created geographical maps with ArcGIS 10.5 and performed the statistical analysis using STATA/IC 13.

### Ethics approval

The study was approved by the ethics committee of the University of Sydney, Australia, and Faculty of Public Health, Universitas Airlangga, Indonesia.

## RESULTS

We mapped a total of 4114 cigarette retailers in Denpasar. The most common type of retailer was a kiosk, 3199 (77.8%), which is a small shop, usually self-owned, which does not require any special permit to establish. Followed by mini markets or convenience stores 606 (14.7%) and 309 (7.5%) were other types of retailers that include wholesalers, supermarkets, mobile phone shops, street vendors (non-movable cart-type vendors), and village/institution co-op.

### Cigarette retailer density in neighbourhoods

Cigarette retailer density in Denpasar was 32.2/km^2^ and 4.6/1000 population. Of the 43 kelurahan/desa, 50% have a tobacco retailer density above 5.2/1000 population and a density of more than 33.9 retailers/km^2^. When taking into account the size of the neighbourhood that is only occupied for housing (excluding farms or fields), half of the neighbourhoods have more than 50 retailer/km^2^ (Table 1). The highest retailer density per population was observed at Kelurahan Ubung, with 12.6 retailers per 1000 population (Supplementary File 1). Some neighbourhoods have an occupied land area less than 0.5 km^2^ resulting in a very high retailer density. For instance, Desa Tegal Kerta, in the sub-district of Denpasar Barat, has a total area of only 0.33 km^2^ and occupied land area of 0.2 km^2^ with a total population of 19998 people; this village then has a retailer density of 5.4/1000 population but an extremely high number density of 330.3 retailers/km^2^ and 495.5 retailers/km^2^ of occupied land size (Supplement File 1).

**Table 1:** Summary of retailer density measures for all neighbourhoods in Denpasar

*Density measure*	*Median (IQR)*	*Min – Max*
Retailers/1000 population	5.2 (4.5–6.2)	1.1 – 12.6
Retailers/km^2^	33.9 (24.7–46.6)	6.4 – 330.3
Retailers/km^2^ occupied land	50.5 (35.0–70.6)	8.7 – 495.5

**Figure 1:**
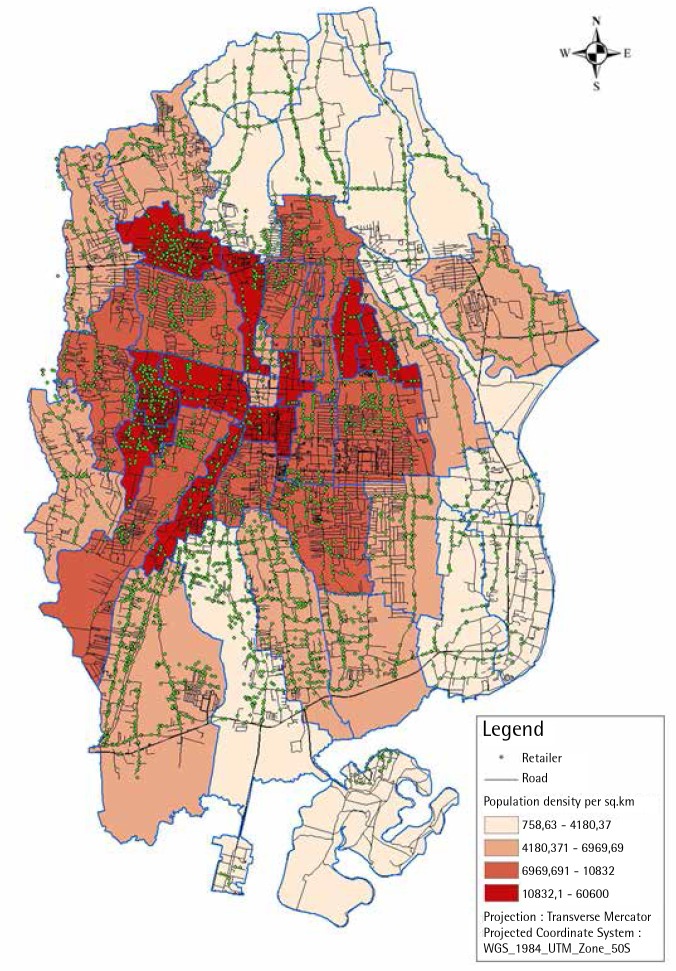
Cigarette retailer distribution based on neighbourhood population density

Retailers are more likely to be present in higher numbers in more populated areas, and the presence of retailers is also related to road access and the presences of housing complexes (Figure 1). Retailers were found in close proximity to other retailers (Table 2), with two-thirds of the outlets having another outlet within 50 m and close to 90% within a 100 m radius. More than 50% of the retailers had more than 3 other retailers within a 100 m radius. A maximum of 8 and 14 outlets were observed within a 50 m and 100 m radius, respectively. On average, there were twelve other outlets within a 250 m radius and thirty-nine other outlets within a 500 m radius, with a maximum of one hundred and thirty-three within 500 m (Table 2).

**Table 2:** Cigarette retailer density and proximity to other retailers in Denpasar (N=4114)

*Distance*	*Number of retailer outlets having at least one other tobacco retailer outlet*		*Number of other outlets around an outlet*	

	*n*	*%*	*Median IQR*	*Min – Max*
Within 50 m radius	2727	66.3	1 (0–2)	0 – 8
Within 100 m	3690	89.7	3 (1–4)	0 – 14
Within 250 m	4096	99.6	12 (7–17)	0 – 53
Within 500 m	4113	99.9	39 (25–55)	0 – 133

### Tobacco retailer density around schools

**Figure 2:**
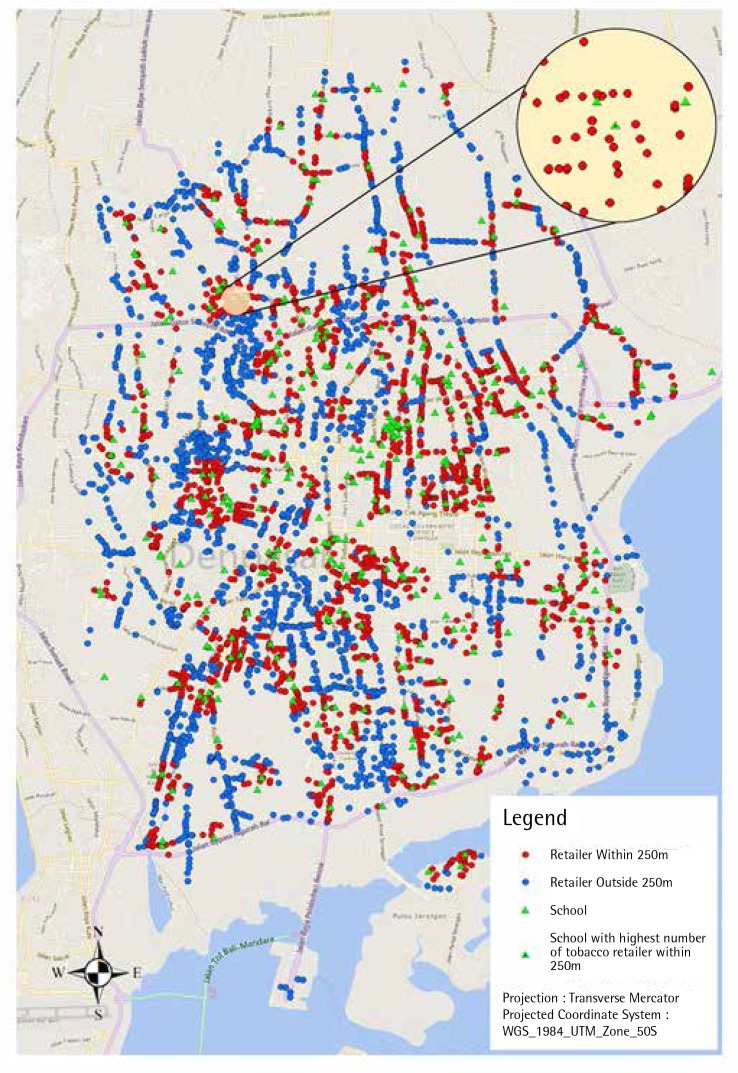
Distribution of cigarette retailers within and outside 250 m radius of schools (inset: a school with 44 outlets within 250 m radius).

Of the 4114 cigarette retailers in Denpasar, 1194 (29.0%) were within a 250 m radius of one or more schools (Figure 2). From a school perspective, of the 379 schools in Denpasar, 9.7% have at least one cigarette retailer within a 25 m radius, two-thirds (68.6%) within a 100 m radius and almost all schools (96.8%) have a tobacco retailer within a 250 m. There is an average of one outlet within a 100 m and nine outlets within a 250 m radius around schools (Table 3). There is one school (a junior high school) with 44 retailers within a 250 m radius and 111 retailers within a 500 m radius (Figure 2). The closest cigarette outlet to a school was only 2.9 m away, and half of the schools had at least one retail outlet within 73.9 m or less (Table 3).

**Table 3:** Density and proximity cigarette retailer around schools in Denpasar (N=379)

*Distance*	*Number of schools having at least one tobacco retailer outlet*		*Number of retailers around a school*	

	*n*	*%*	*Median IQR*	*Min – Max*
Within 25 m radius	37	9.8	0 (0–0)	0 – 3
Within 50 m	120	31.7	0 (0–1)	0 – 6
Within 100 m	260	68.6	1 (0–3)	0 – 11
Within 250 m	367	96.8	9 (5–14)	0 – 44
Within 500 m	376	99.2	34 (21–49)	0 – 111
Distance to the closest retailer (m)			73.9 (42.9–114.0)	2.9 – 697.8

Based on school level and type, there is not much difference in the proportion of schools that have at least one cigarette outlet within a certain radius. Primary schools are more likely to have a retail outlet within a 25 m and 50 m radius, compared to other school levels (Table 4).

**Table 4:** Presence of at least one cigarette outlet within a certain radius based on the school’s type and level

*School characteristics*	*Number of schools with at least one cigarette outlet within each radius (m) n (%)*				

	*25*	*50*	*100*	*250*	*500*
**School level**					
Primary school (N=239)	31 (13.0)	88 (36.8)	166 (69.5)	230 (96.2)	237 (99.2)
Junior high school (N=69)	4 (5.8)	18 (26.1)	27 (39.1)	66 (95.7)	68 (98.6)
Senior high school (N=71)	2 (2.8)	14 (19.7)	48 (67.6)	71 (100)	71 (100)
p*	0.02	0.01	0.75	0.25	0.62
**School type**					
Public (N=193)	22 (11.4)	68 (35.2)	134 (69.4)	191 (99.5)	193 (100)
Private (N=186)	14 (7.7)	52 (28.0)	126 (67.7)	176 (94.6)	183 (98.4)
p*	0.27	0.13	0.72	<0.01	0.08

* Based on chi-squared test.

### Retailer selling practices

Based on the audit survey data of 1000 selected retailers, 281/485 (57.9%) of the audited retailers within 250 m of a school admitted selling cigarettes to young people, and 325/485 (67.0%) said they sold cigarettes as single sticks (Table 5). Retailers that were a further distance from schools were less likely to sell cigarettes to young people, though the only significant difference was between the retailers at more than 500 m from school compared to those at a 100 m radius (Table 5). Meanwhile, there was no significant difference in selling single sticks based on retailer distance from schools. Retailer type is a significant predictor of selling single sticks (Table 5), mini markets (AOR=0.06, p<0.001) and other types of retailers (AOR=0.09, p<0.001) are much less likely to sell ‘loosies’ when compared to kiosks. Other retailer types are also 54% less likely to sell to young people than kiosks (AOR=0.47, p=0.014).

**Table 5:** Retailer behaviour based on its proximity to school

*Predictor*	*Selling to young people*			*Selling single stick*		

	*n (%)*	AOR* (95% CI)	*p*	*n (%)*	AOR* (95% CI)	*p*
**Distance from school**						
≤100 (N=144)	87 (60.4)	Ref.		94 (65.2)	Ref.	
100.1–250 (N=341)	194 (56.9)	0.84 (0.56–1.26)	0.4	231 (67.7)	1.14 (0.71–1.83)	0.59
250.1–500 (N=388)	203 (52.3)	0.70 (0.47–1.04)	0.08	228 (58.8)	0.69 (0.44–1.10)	0.12
>500 m (N=127)	57 (44.9)	0.53 (0.33–0.87)	0.01	72 (56.7)	0.61 (0.35–1.08)	0.09
**Level of school closest to the retailer**						
Primary (N=723)	389 (53.8)	Ref.		453 (62.7)	Ref.	
Junior high school (N=147)	79 (53.7)	0.88 (0.60–1.30)	0.52	97 (66.0)	1.02 (0.64–1.62)	0.93
Senior high school (N=129)	73 (56.6)	0.94 (0.62–1.43)	0.78	75 (58.1)	0.69 (0.42–1.11)	0.12
**Type of school closest to the retailer**						
Public (N=632)	329 (52.1)	Ref.		387 (61.2)	Ref.	
Private (N=368)	212 (57.6)	1.29 (0.95–1.74)	0.1	238 (63.7)	1.28 (0.90–1.82)	0.17
**Type of retailer**						
Kiosk (N=800)	438 (54.8)	Ref.		592 (74.0)	Ref.	
Mini market (N=151)	85 (56.3)	1.06 (0.73–1.49)	0.75	23 (15.2)	0.06 (0.04–0.97)	<0.001
Others (N=49)	18 (36.7)	0.47 (0.25–0.84)	0.01	10 (20.4)	0.09 (0.04–0.18)	<0.001

* AOR: adjusted odds ratio.

Adjusted for all variables in the models: school distance, school level, school type and retailer type.

## DISCUSSION

Retailers are the public face of the tobacco marketing and distribution chain. The physical presence of cigarette outlets is a significant barrier to denormalising smoking, especially amongst young people where retail outlets enhance perceived availability^6^ and foster exposure to tobacco promotion^40^. Our study paints a portrait of cigarette retailer distribution in an Indonesian city, where cigarette retailing is almost entirely unregulated. We found a high density of cigarette outlets within a close distance to schools. While there was a trend in being less likely to sell cigarettes to young people based on distance from schools, this was only significant at the furthest distance of more than 500 m from schools. Selling tobacco to youth is commonplace and minimally discouraged, regardless of where the outlet is located.

In a poorly regulated retailing system such as Indonesia, we can expect that cigarette retailers are as ubiquitous as found in the city of Denpasar. The tobacco retailer density of 4.6 per 1000 people is 20 times higher than the retailer density found in New South Wales, Australia, at 0.22 per 1000 in 2014^16^, and also higher compared to the maximum cap of 3.5 per 1000 adopted in China, a nation with a state-owned tobacco company monopoly^29^.

Our findings highlight the high availability of cigarette retailers around schools and in neighbourhoods. Cigarette retailers were found as close as 2.9 m from schools, and around 10% of the schools had at least one retailer within a 25 m radius. Proximity to primary schools was highest, and this may be because these schools are usually closer to neighbourhood living areas compared to other school levels. Primary school students are also vulnerable to smoking, with 19.8% of Indonesian student smokers starting to smoke before they are 10 years old^24^.

Unsurprisingly, a higher number of cigarette outlets were observed in the more populated areas of Denpasar, and most of these retailers were small self-owned kiosks. Establishing a kiosk business is extremely easy in Indonesia, as small self-owned enterprises do not require any type of permit^41^, nor are they required to be in a specific location. For example, a kiosk that sells cigarettes can be opened out of a home garage. The excessive presence of cigarette retailers signifies that cigarettes are easy to access and has been shown to influence young people’s smoking behaviour^9,10,42^.

The notion of easy access was further emphasized by our finding that kiosk retailers are significantly more likely to sell to young people and to sell single sticks compared to other retailers. GYTS 2014 showed that more than half (63.2%) of student smokers aged 13–15 years purchased their cigarettes in a shop or from a kiosk^24^. We found a trend that retailers are less likely to sell cigarettes to young people and to sell single sticks if further away from schools, but this was only statistically significant at distances >500 m. These data show that young people are essentially able to access cigarettes anywhere, including around education institutions. Selling cigarettes to young people is an accepted and common practice in Indonesia, as evidenced by the lack of enforcement on the prohibition of selling to young people below 18 years^23^.

Besides being a sign of easy access, high retailer density also contributes to high exposure to tobacco marketing, as tobacco advertising and promotion (TAPS)^33^ at point-of-sale is yet to be prohibited in Denpasar^35^, and in most other Indonesian cities. These environmental factors are significant factors in the rising smoking prevalence among Indonesian youth^25,43^.

Several approaches are available for regulating tobacco retailer density, including licensing, zoning, proximity limits, and capping the number of retailers^26-28,44^. Tobacco products are acknowledged as an addictive substance in The Indonesian Health Law no 36/2009^45^, which should mean that cigarettes are strictly regulated — similar to alcohol and other harmful drugs. While alcohol premises must be licensed^46^, selling cigarettes is unregulated even in the face of rising smoking prevalence^43,47^ and increasing death toll^48^. The discrepancy between cigarette and alcohol sales regulations may be influenced by religious prohibitions on drinking alcohol and the more immediate social effects of alcohol consumption. Investigating if alcohol licensing procedures and rationale could be applied to cigarette retailing warrants further examination.

The introduction of a tobacco-licensing scheme, that includes a permit fee, could reduce cigarette retailers by up to one-third^19^. Licensing would also benefit local governments by providing a source of funds to support improved monitoring and enforcement of tobacco retailing laws^49^. The impact of a licensing system could be even more significant in Indonesia, where nearly 80% of tobacco retailers are small, low-revenue kiosks. These small, low volume, retailers may be more likely to opt out of cigarette retailing in order to avoid licensing procedures and fees. Tobacco company incentives may be offered to try and counter this effect^50,51^, but there is currently no available evidence on Indonesian small retailer reliance on cigarette sales. A UK study reported almost 90% of tobacco retailers in the disadvantaged areas of Newcastle and London reported a low-profit margin from selling cigarettes but perceived a high reliance on cigarette sales as customers who bought cigarettes were also buying other products^52^.

A licensing scheme could be paired with the adoption of a retail zoning scheme, such as prohibiting cigarette retailers around educational institutions^44^. Zoning is also a possible stand-alone option if establishing a licensing scheme in Indonesia is too premature or complicated. Zoning may also be a more feasible approach in the Indonesian system of decentralised government. Sub-national governments have the authority to introduce retailer zoning as part of city planning^53,54^. The adoption of zoning regulations aligns with existing smoke-free bylaws and the establishment of child-friendly cities. There are several variations on the distance to tobacco retailer prohibitions around schools, such as within 100 yards (90 m) in India^28^, within 100 m in China^26^, and within 500 feet (152.4 m) in San Francisco^27^. Based on our findings, adopting a 500 m zoning in Denpasar may deliver the greatest impact, but the adoption of an at least 100 m radius will likely reduce youth exposure to cigarette marketing.

A further regulatory approach is to decrease retailer density by limiting proximity between retailers and capping the maximum number of retailers in a particular jurisdiction^44^. These approaches (zoning and capping) are usually attached to a licensing scheme for current and new cigarette retailers. San Francisco has adopted such comprehensive measures that include capping the total number of retailers per supervisorial district to 45, prohibiting cigarette retailing within 500 feet of schools and within 500 feet of another retailer, and denying permits for new tobacco retailers^27^.

These retailing regulatory frameworks to control the supply side of tobacco consumption could be another way to complement existing tobacco control measures in Indonesia. Adopting proven demand reduction measures, including raising cigarette taxes, must also remain a national priority^55^. Despite slow progress at the national level, some sub-national governments have demonstrated support for tobacco control initiatives through the adoption of smoke-free public places^56^, limited TAPS bans, and tobacco display bans at retail^57-59^. Measures to regulate retailers could be a next step for cities/districts with more advanced tobacco control policies already in place.

### Limitations

To our knowledge, this is the first paper to map cigarette retailer distribution in an unregulated setting. However, our study has some limitations. First, our estimate is likely to be lower than the actual number of outlets, as we excluded hotels, restaurants and bars from the study. Cigarette purchases in these other venues are likely limited to patrons and do not reflect general public access. Second, although we found a higher number of retailers in the more populated areas, unfortunately, we were unable to show the disparity of retailer density based on socioeconomic status due to limited available data. Third, the geographical mapping unit was only available at kelurahan/desa level, which may not be the best unit to show differences in density since it is a large area with a relatively wide socioeconomic variation. This lack of availability of quality secondary data is a common challenge for researchers in low-and middle-income settings.

## CONCLUSIONS

Our findings suggest that cigarettes are highly available and accessible by young Indonesians both in their neighbourhoods and in areas around schools. Regulating cigarette retailers is another pillar of effective tobacco control that should be implemented by the Indonesian government, alongside other proven demand reduction measures. For some cities/districts with more progress in regulating smoke-free areas and TAPS bans, this measure could be the next stage in strengthening tobacco control. Enforcing the prohibition of selling tobacco to minors must also be a priority. Further studies should examine smoking behaviour in connection to retailer density around schools and in neighbourhoods, retailer reliance on income from cigarette selling, and retailer and stakeholder perspectives on strengthening retail regulation. We also recommend replication of similar mapping studies in other Indonesian cities to build stronger evidence for policy adoption.
