# Supplementary material for: Cigarette retailer density around schools and neighbourhoods in Bali, Indonesia: A GIS mapping
Source: Tob Induc Dis. 2019 Jul 5;17:55. doi: 10.18332/tid/110004 (PMC6770614; doi:10.18332/tid/110004)
Supplement: Supplementary file 1 [file TID-17-55-s1.pdf]

Supplement 1.
Population and cigarette retailer density of all desa/kelurahan in Denpasar

Sub-District	Desa / Kelurahan	Total Area (km2)	Occupied land (km2)	Total Population	Population density/ km2	No of retailer	Retailer density/ 1000 peoples	retailer density/ km2	retailer density/km2 of occupied land
Denpasar utara	Dangin Puri Kaja	1.42	1.26	13,824	9735.21	67	4.85	47.18	53.17
Denpasar utara	Dangin Puri Kangin	0.75	0.71	8,124	10832.00	40	4.92	53.33	56.34
Denpasar utara	Dangin Puri Kauh	0.72	0.66	3,661	5084.72	23	6.28	31.94	34.85
Denpasar utara	Paguyangan	6.44	4.91	15,191	2358.85	109	7.18	16.93	22.20
Denpasar utara	Paguyangan Kaja	5.36	2.80	7,850	1464.55	75	9.55	13.99	26.79
Denpasar utara	Paguyangan Kangin	4.16	1.82	16,433	3950.24	106	6.45	25.48	58.34
Denpasar utara	Tonja	2.3	2.12	19,723	8575.22	107	5.43	46.52	50.47
Denpasar utara	Ubung*	1.03	0.91	11,988	11638.83	151	**12.60**	146.60	166.59
Denpasar utara	Ubung Kaja	4.3	3.04	25,761	5990.93	140	5.43	32.56	46.01
Denpasar utara	Dangin Puri Kelod	2.09	1.18	15,661	7493.30	78	4.98	37.32	66.10
Denpasar utara	Dauh Puri Kaja	1.09	0.99	14,965	13729.36	48	3.21	44.04	48.48
Denpasar utara	Pamecutan Kaja	3.85	3.66	38,379	9968.57	199	5.19	51.69	54.37
Denpasar Barat	Dauh Puri	0.6	0.57	9,067	15111.67	20	2.21	33.33	35.09
Denpasar Barat	Dauh Puri Kangin	0.59	0.46	3,597	6096.61	4	1.11	6.78	8.70
Denpasar Barat	Dauh Puri Kauh	1.83	1.79	21,649	11830.05	91	4.20	49.73	50.84
Denpasar Barat	Dauh Puri Kelod	1.88	1.62	15,132	8048.94	79	5.22	42.02	48.77
Denpasar Barat	Padangsambian	3.7	2.78	35,666	9639.46	134	3.76	36.22	48.20
Denpasar Barat	Padangsambian Kaja	4.09	2.73	20,499	5011.98	101	4.93	24.69	37.00
Denpasar Barat	Padangsambian Kelod	4.12	2.65	23,871	5793.93	85	3.56	20.63	32.08
Denpasar Barat	Pamecutan	1.86	1.76	21,099	11343.55	70	3.32	37.63	39.77
Denpasar Barat	Pamecutan Kelod	4.42	3.92	45,552	10305.88	132	2.90	29.86	33.67
Denpasar Barat	Tegal Harum	0.62	0.21	13,304	21458.06	69	5.19	111.29	328.57
Denpasar Barat	Tegal Kertha**	0.33	0.22	19,998	60600.00	109	5.45	**330.30**	**495.45**
Denpasar Timur	Kesiman	2.39	0.78	14,960	6259.41	64	4.28	26.78	82.05

Denpasar Timur	Kesiman Kertalangu	3.8	2.36	26,037	6851.84	110	4.22	28.95	46.61
Denpasar Timur	Kesiman Petilan	2.81	2.16	11,525	4101.42	68	5.90	24.20	31.48
Denpasar Timur	Penatih	2.91	0.94	11,188	3844.67	73	6.52	25.09	77.66
Denpasar Timur	Penatih Dangin Puri	3.12	0.55	6,894	2209.62	55	7.98	17.63	100.00
Denpasar Timur	Sumerta	0.5	0.47	10,210	20420.00	51	5.00	102.00	108.51
Denpasar Timur	Sumerta Kaja	0.52	0.20	8,330	16019.23	59	7.08	113.46	295.00
Denpasar Timur	Sumerta Kauh	0.87	0.85	7,668	8813.79	42	5.48	48.28	49.41
Denpasar Timur	Sumerta Kelod	2.68	2.47	19,133	7139.18	125	6.53	46.64	50.61
Denpasar Timur	Dangin Puri	0.62	0.61	6,798	10964.52	21	3.09	33.87	34.43
Denpasar Selatan	Panjer	3.59	2.52	36,665	10213.09	145	3.95	40.39	57.47
Denpasar Selatan	Pedungan	7.49	3.95	31,311	4180.37	250	7.98	33.38	63.32
Denpasar Selatan	Pemogan	9.71	4.73	46,372	4775.70	353	7.61	36.35	74.61
Denpasar Selatan	Renon	2.54	1.26	17,703	6969.69	89	5.03	35.04	70.56
Denpasar Selatan	Sanur	4.02	3.57	14,868	3698.51	60	4.04	14.93	16.79
Denpasar Selatan	Sanur Kaja	2.69	1.76	8,957	3329.74	48	5.36	17.84	27.26
Denpasar Selatan	Sanur Kauh	3.86	2.09	14,628	3789.64	73	4.99	18.91	34.99
Denpasar Selatan	Serangan	4.81	0.22	3,649	758.63	31	8.50	6.44	138.45
Denpasar Selatan	Sesetan	7.39	4.56	50,303	6806.90	262	5.21	35.45	57.45
Denpasar Selatan	Sidakarya	3.89	2.41	20,395	5242.93	98	4.81	25.19	40.63

*) Desa/kelurahan with the highest retailer density/population

**) Desa/kelurahan with the highest retailer density/km^2

© 2019 Astuti P.A.S.
